# The Effectiveness of Mating Induction on Men’s Financial Risk-Taking: Relationship Experience Matters

**DOI:** 10.3389/fpsyg.2021.787686

**Published:** 2022-01-11

**Authors:** Tingting Liu, Zhuanzhuan Wang, Anrun Zhu, Xi Zhang, Cai Xing

**Affiliations:** Department of Psychology, Renmin University of China, Beijing, China

**Keywords:** mating motivation, risk-taking, relationship experience, evolutionary psychology, mating induction

## Abstract

Substantial evidence from experimental studies has shown that mating motivation increases men’s financial risk-taking behaviors. The present study proposed a new moderator, men’s past relationship experience, for this well-accepted link between mating motivation and financial risk-taking tendency. Heterosexual young men were randomly assigned to the mating condition and control condition, and they completed a set of financial risk-taking tasks and reported their past relationship experience. A significant main effect of mating motivation and a significant interaction effect between experimental conditions (*mating* group and *control* group) and relationship experience emerged, suggesting that mating motivation increased financial risk-taking tendency only among men who have never been committed in a romantic relationship, rather than those who have had such experience. This moderating effect was replicated in two experiments. The present study contributed to the understanding of individual differences in the relationship between mating motivation and male financial risk-taking. The present findings also have important implications for financial industry and gambling companies to better target clients and advertise their high-risk products.

## Introduction

“Water is hardly water without ocean,

cloud is hardly cloud without mountain,

worldly beauty no longer appeals to my eyes, due to my love to you.”

From the Chinese ancient poem “Li Si” by Yuan Zhen.

The famous poem of ancient Chinese poet Yuan Zhen for commemorating his deceased wife has been favored for thousand years reflecting that, in Asia, people endorse men with their past relationship experience. Similarly, in the Western pop culture, there is a famous comedy, The 40-year-old Virgin tells a story that a 40-year-old sexually inexperienced man was mocked and ridiculed by his colleagues and his friends helped him to find a true love. This movie reveals the other side that not having relationship experience decreases men’s mating value. Recent research in mate choices and preference implied that women similarly prefer romantically experienced men as their mate (see review in Anderson, 2018), it is still unclear what role of men’s relationship experience plays in men’s mating process. The focus of the present study is to examine if relationship experience moderated the effect of mating induction on men’s financial risk-taking.

### Mating Motivation, Sexual Selection, and Financial Risk-Taking

Evolutionary theories and research have found that some specific behaviors are evolved as a result of sexual selection (Trivers, 1972; Darwin, 1979; Griskevicius et al., 2006; Eastwick et al., 2014). To be more specific, as the result of the intrasexual selection, a competition among same-sex for mate access, males would evolve weaponry attributes, such as larger body size; while as the result of the intersexual selection, a process of one sex (usually females) choose the opposite sex member as a mate, males would evolve ornament-like attributes that would be preferred by females (Chang et al., 2011; Chen and Chang, 2015; Lu et al., 2015; Liu and Xing, 2015; Liu, 2020). One of these behavioral consequences from intersexual selection is the risk-taking behaviors among men (Xing et al., 2015). According to the Costly signaling theory (CST, Zahavi, 1975; Grafen, 1990), through risk-taking, men could send costly but honest signals reflecting traits of a good mate for women, such as ambition, social dominance, and confidence, to the potential mate targets (Xing et al., 2015). These traits have been shown to be preferred by women in their mate choices (Shackelford et al., 2005; Schmitt, 2005, 2014; Lu et al., 2015). Empirical findings also evidenced that women indeed favor risk-taking men over risk-averse men as romantic partners (Bassett and Moss, 2004; Farthing, 2005, 2007). This means that men could increase their chance of winning the intersexual selection and increase their reproductive success through risk-taking (Baker and Maner, 2009). Due to the sex difference in mate choice and preference, in the intersexual selection process, risk-taking benefits men more, than women, and is always considered as a male mating strategy (Buss, 1989), especially in the context of the Chinese culture (Shan and Jin, 2013). For example, research using Chinese sample found that mating-related after-task evaluations motivated Chinese male to be riskier but motivated Chinese females to be more risk-aversion in the Balloon Analogue Risk Task (Shan et al., 2012).

Among all risk-taking behaviors, researching financial risk-taking has implications for everyday life as it evaluates individuals’ choice of risky outcomes with direct monetary rewards, and this positive link between mating goal and men’s financial risk-taking has been widely demonstrated in the past research (e.g., Van Den Bergh et al., 2008; Greitemeyer et al., 2013; Herbert, 2018). For example, heterosexual men were found to become more risk-seeking on financial tasks after viewing erotic pictures (Knutson et al., 2008). Another study showed that exposure to highly attractive women increased men’s financial risk-taking, whereas exposure to unattractive women imposed no effect on men’s risk-taking (Baker and Maner, 2008). Based on this, Baker and Maner (2009) later found that men became riskier in a risk-taking task when they were expecting to interact with an attractive female confederate.

### Men’s Relationship Experience as a Potential Moderator

In the process of intersexual selection, women’s mate choice is important and is more discerning (Anderson and Surbey, 2020). While many studies have addressed what impacts women’s mate choice, some initial discussions suggest that the mate or mate choice copying phenomenon, a phenomenon well-evidenced in non-human species showing that females’ mate choices are made with information from other females and they should prefer mated male over the unmated male in mating, also occurs among humans (see review in Anderson, 2018). This reflects that women’s mate selection is not independent but a process resulting from social learning (Zhuang et al., 2016; Anderson and Surbey, 2020). Some initial empirical evidence has proved the existence of this phenomenon in human females and women prefer mated man over unmated man in mating selection (Anderson and Surbey, 2014; Scammell and Anderson, 2020). For example, Anderson and Surbey (2014) asked women to rate men pictured with none, one, two, or five women representing having no romantic relationship in the past 4 years, or have been one, two or five previous partners; they found that men with one or two previous partners were rated to be more desirable as compared to that of men with no previous romantic relationship in the past 4 years. Stewart-Williams et al. (2017) also found that females’ average willingness to engage in a romantic relationship with a hypothetical man increases as his past partner number increases and reached the peak of two or three previous partners.

On the basis of this, we suggested that mating-induced male risk-taking may depend on individual differences in men’s romantic relationship experience. Women’s mate choice shapes men’s traits in the long term of evolution (Buss, 1989). That is, for a man, traits or behaviors that are in line with females’ mate preference should be encouraged if mating goals are activated (Chang et al., 2011). As women are found to prefer romantically experienced men, men with no past romantic relationship should exhibit more other desirable traits to fit into women’s preference in the context of mating. Then, unmated man should have stronger motivation to involve in financial risk-taking after priming mating motives, to increase their opportunity in mating market. This is similar to the desperado effects observed in males’ aggression behaviors, where males with disadvantage in mating would fight harder and have stronger motives to seek conflicts with stronger same-sex members because they have nothing to lose (Grafen, 1987).

On the other side, the experience of having a romantic partner is considered as a reproductive success. Men who have had successful mating experience in their mate search process perceive a higher level of social dominance, they have a bigger chance of winning the intersexual selection as they are preferred by women. Involving in such financial risk-taking behaviors might generate more costs than receive benefits for them. That is, mated men have less motivation to involve in financial risk-taking after priming mating motives. Consistent with this idea it has been suggested that the effect of the mating prime on men could be eliminated when men achieved dominance over other men (Ainsworth and Maner, 2012). Similar findings observed in the non-human species, for example, successfully mated male field crickets (*Orthoptera: Gryllidae: Gryllinae*) were less likely than unmated males to win in the male-male aggressive contest (Judge et al., 2010).

### The Current Study and Hypotheses

The focus of the present study is to examine if men’s romantic relationship experience will moderate the effect of mating motivation on men’s financial risk-taking. It was hypothesized that for men who have never been in a romantic relationship, mating motivation induction would lead to more significant increase in men’s risk-taking propensity than those who have had the experience of committing to a romantic relationship. Across two experiments, we tested this possibility by inducing mating motivation among men with and without romantic experiences. It was also expected that the present study would replicate the past finding that mating induction led to increased willingness to take risks among men.

## Experiment 1

### Method

#### Participants and Design

Participants were 90 heterosexual men (*M*_age_ = 22.89, *SD*_age_ = 3.10, age range 19–34). They were college students recruited from a university in Beijing, China, as an exchange for course credits. Participants were randomly assigned to one of the two experimental conditions: *mating* condition vs. *control* condition.

#### Mating Induction

Participants in both conditions viewed and rated five photographs, ostensibly in preparation for future studies. Participants in the *mating* condition viewed and rated five photographs of five attractive young women (an established technique to activate mating motivation, Baker and Maner, 2008; Lu and Chang, 2012) and were asked to choose one woman they wanted to date most; whereas participants in the *control* condition rated five pictures of natural scenery and indicated which place they wanted to visit most. All ratings were made on a scale from 1 (not attractive at all) to 9 (extremely attractive). A pilot rating (*N* = 63, *M*_age_ = 22.23) confirmed that the photographs of attractive women were significantly above average in desirability, *M* = 6.36, *SD* = 1.13, *t* (62) = 9.55, *p* < 0.001.

#### Measures

##### Financial Risk-Taking

Participants answered ten investment questions which served as an indicator of their financial risk-taking tendency. They indicated on a five-point scale their preference between a safe option and a risky option, where 1 indicated absolutely prefer the safe option and 5 indicated absolutely prefer the risky one (α = 0.83). All of the safe options offered 100% chance to gain a certain amount of money, whereas the risky options offered 5 to 95% chance to gain more money. The expected utilities were equivalent between the safe option and the risky option. As an example, one of the 10 questions provided two options for the participants to choose: the safe option offered 100% chance to gain 80 Chinese Yuan (1 Yuan = 0.163 USD), while the risky option offered 80% chance to gain 100 Chinese Yuan. Five out of the ten investment questions were reverse coded. Participants’ sum scores on these 10 questions served to indicate their financial risk-taking tendencies. This set of questions has been used among Chinese participants as a measure of risk-taking (Liu et al., 2010).

##### Past Relationship Experience and Current Relationship Status

Participants reported their relationship experiences by answering the question: “whether you had been committed to a serious romantic relationship?” They were also asked to report their current relationship status (“whether you are in a relationship now?”), because these two variables may be related and impact the dependent variable. Given the fact that there is not a unified standard to answer these two questions, participants were allowed to judge their current relationship status and relationship experiences by their own standard (i.e., no restrictions were placed as to how to define their relationship status and experience).

#### Procedure

To dissociate the mating induction and financial risk-taking task, participants were told that they were going to participate in two unrelated experiments. Because both experiments were very short, for efficiency, the two experiments would be completed together in one experiment. This cover story was adapted from previous research (Griskevicius et al., 2007). Then, participants started to work on the first experiment, during which they went through the mating induction procedure. After the mating induction, the participants were instructed to finish the second experiment, which was actually designed to examine the effect of mating induction on risk-taking tendencies. Finally, participants reported their demographic information, in which they indicated their current relationship status and their relationship experience.

### Results

#### Preliminary Analysis

SPSS was used for data analysis. A univariate ANOVA was conducted, experimental condition and relationship experience and relationship status served as the independent (between-subject) variables, and participants’ average scores on the investment questions served as the dependent variable. No main effect of current relationship status was found on participants’ risk-taking tendencies (*N*_single_ =, *M*_single_ = 2.87, *SD*_single_ = 0.82; *N*_in a relationship_ = *M*_in a relationship_ = 2.76, *SD*_in a relationship_ = 0.84), *F*(1,82) = 0.79, *p* = 0.377, neither was there any interaction effect between relationship status and the other two independent variables on risk-taking, *F*(1,82) = 0.39, 0.28 and 0.77 for the three-way interaction, *p*s > 0.384. Therefore, data were collapsed across current relationship status.

Preliminary analysis confirmed that participants in the four experimental conditions were generally equivalent in terms of their economic situation (i.e., their self-reported monthly expenses). Specifically, participants in the *mating* condition (*M* = 2.87, *SD* = 1.23) and *control* condition (*M* = 3.03, *SD* = 1.11) did not differ regarding their economic situation, *t*(88) = −0.62, *p* = 0.538. Neither did participants with relationship experience (*M* = 2.85, *SD* = 1.23) and those without relationship experiences (*M* = 2.98, *SD* = 1.16) differed in terms of their monthly expenses, *t*(88) = 0.52, *p* = 0.606.

#### Hypothesis Testing

**Figure 1:**
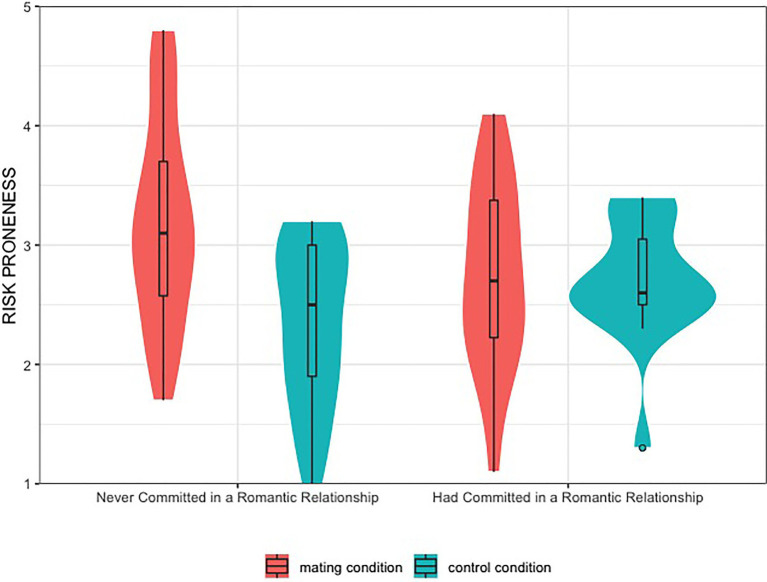
Men’s financial risk-taking tendency as a function of condition and relationship experience in Experiment 1.

A univariate ANOVA was conducted with experimental condition and relationship experience as independent (between-subject) variables, participants’ average scores on the investment questions served as the dependent variable, age, economic situation and relationship status were controlled in the analysis. As expected, participants in the *mating* condition (*M* = 3.04, *SD* = 0.86) were more willing to take risks than those in the *control* condition (*M* = 2.49, *SD* = 0.64), *F*(1,83) = 6.33, *p* = 0.014, $\eta_{p}^{2}$ = 0.071. Participants with relationship experience (*M* = 2.71, *SD* = 0.68) did not differ from those without relationship experiences (*M* = 2.88, *SD* = 0.90), *F*(1,83) = 0.44, *p* = 0.508. This finding was qualified by a significant interaction effect between experimental condition and relationship experience, *F*(1,83) = 5.63, *p* = 0.020, $\eta_{p}^{2}$ = 0.064. Follow-up simple effects analyses showed that, as predicted, male participants who had never committed in a serious romantic relationship showed higher risk-taking tendencies after their mating motivation was induced (*mating*: *M* = 3.19, *SD* = 0.86, *control*: *M* = 2.34, *SD* = 0.68), *F*(1,83) = 15.77, *p* < 0.001, $\eta_{p}^{2}$ = 0.160; in contrast, mating induction did not make a significant difference in participants’ risk-taking tendencies for those who had been involved in a serious relationship (mating: *M* = 2.73, *SD* = 0.79, control: *M* = 2.69, *SD* = 0.53), *F*(1,83) = 0.01, *p* = 0.922. See Figure 1.

## Experiment 2

One limitation of Experiment 1 was the small sample size. Therefore, we conducted a replication study with a larger sample size, to test the robustness of the findings in Experiment 1.

### Method

#### Power Analysis

A power analysis using G*Power (Faul et al., 2009) was performed. The effect size coefficient of $\eta_{p}^{2}$ = 0.160 was used from study 1. Power analysis indicated that study 2 required 322 participants to obtain a power of 0.95 with an alpha of 0.05.

#### Participants

Three hundred and seventy-two male participants were recruited from China. Participants who failed the attention check (*N* = 32) were excluded from data analyses. This left a final sample of 340 participants (*M*_age_ = 22.09, *SD*_age_ = 2.71, age range 18–30). They were randomly assigned to one of the two experimental conditions: mating condition vs. control condition.

#### Measures and Procedure

The measures and procedure of Experiment 2 were the same as in Experiment 1, except that Experiment 2 was collected through an online crowdsourcing platform in China, powered by www.wjx.cn., and two attention check questions were added.

### Results

First, we examined participants’ economic situation (i.e., their self-reported monthly expenses) across conditions. As in Experiment 1, participants in the *mating* condition (*M* = 4.83, *SD* = 1.49) and *control* condition (*M* = 4.75 *SD* = 1.79) did not differ regarding their economic situation, *t* (388) = −0.45, *p* = 0.654. Neither did participants with relationship experience (*M* = 4.85, *SD* = 1.62) and those without relationship experiences (*M* = 4.74, *SD* = 1.67) differed in terms of their monthly expenses, *t*(311) = −0.594, *p* = 0.553 Economic situation was entered as a covariate in the subsequent analyses.

**Figure 2:**
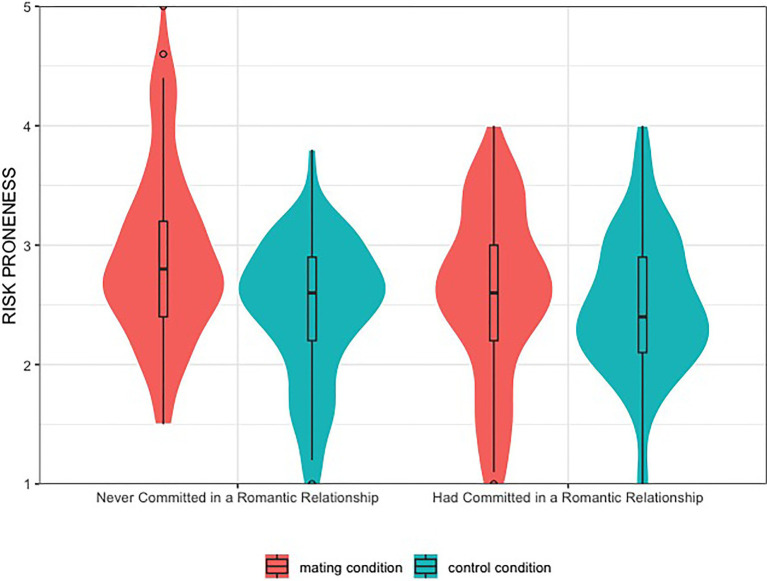
Men’s financial risk-taking tendency as a function of condition and relationship experience in Experiment 2.

Next, a univariate ANOVA was conducted with experimental condition and relationship experience as independent (between-subject) variables, participants’ average scores on the investment questions served as the dependent variable, age, economic situation, and relationship status were controlled in the analysis. Consistent with study 1, participants in the *mating* condition (*M* = 2.75, *SD* = 0.75) were more willing to take risks than those in the *control* condition (*M* = 2.48, *SD* = 0.59), *F*(1,339) = 14.10, *p* = 0.000, $\eta_{p}^{2}$ = 0.041. Participants without relationship experiences (*M* = 2.69, *SD* = 0.71) showed a higher risk proneness than those with relationship experiences (*M* = 2.54, *SD* = 0.66), *F*(1,339) = 6.87, *p* = 0.009, $\eta_{p}^{2}$ = 0.020. As expected, a two-way interaction emerged between the two independent, *F*(1,339) = 7.78, *p* = 0.006, $\eta_{p}^{2}$ = 0.023. Follow-up simple effects analyses showed that, as predicted, male participants who had never committed in a romantic relationship showed higher risk-taking tendencies after their mating motivation was induced (*mating*: *M* = 2.93, *SD* = 0.07, *control*: *M* = 2.46, *SD* = 0.07), *F*(1,336) = 20.84, *p* = 0.000, $\eta_{p}^{2}$ =0.058; in contrast, mating induction did not make a significant difference in participants’ risk-taking tendencies for those who had been involved in a romantic relationship (mating: *M* = 2.58, *SD* = 0.07, control: *M* = 2.49, *SD* = 0.07), *F*(1,336) = 0.78, *p* = 0.376. See in Figure 2.

## Discussion

With two experiments, the present work replicated an important and well-documented finding in the field of evolutionary psychology, such that inducing mating motivation would lead to increased financial risk-taking (Baker and Maner, 2008, 2009; Van Den Bergh et al., 2008; Geniole and Carré, 2018). Moreover, the present study further showed that mating-related increases in financial risk-taking among men were specific to those who had never been in a romantic relationship. For the men who had been in a romantic relationship, the effect of the mating prime was eliminated.

The current findings demonstrated the importance of men’s past romantic relationship experience in the mating selection. Though past studies suggest that women prefer men with some romantic relationship experience (Anderson, 2018), our current study provides this from men’s side. Confirming the findings in animal research and aggression studies (e.g., Judge et al., 2010), we showed that men with no relationship experience displayed stronger risk-taking tendency when making financial decisions in the context of mating. According to mate copying theory, mated man should be more favored by women in mate choice (Anderson and Surbey, 2014; Scammell and Anderson, 2020). This makes these mated men, as compared to these unmated men, enter the situation of having advantage in intersexual selection when mating goals are highlighted in our task, which might eliminate the effect of the mating prime subsequent financial risk-taking tasks. Considering the risk-taking behaviors are situational male mating strategy (Baker and Maner, 2008), we believe that not having past romantic relationship experience enhances this mating strategy in men.

Results from the present study might also offer an additional explanation to the well-documented Young Male Syndrome, which occurs in human males during the late teen and early 20s (Wilson and Daly, 1985), and indicated that young adults more than any other age group engage in risky behavior, and age difference in risk-taking is especially pronounced at early adulthood (Turner and McClure, 2003; Steinberg, 2008). Past explanation for this risk-taking tendency was that young men are highly motivated to gain sexual access to women although they are less likely to attract highly fertile women than older men, therefore the utility of male risk-taking is greatest for young men (Barber, 2009a,b, 2011) as a way to exhibit their advantages, such as physical fitness and courage. Our present study supported the utility account from a new perspective It might be possible that, compared with older men, young men in their late teen or early 20s lack romantic relationship experience and have stronger motivation to take the risks to attract women (Barber, 2009a,b).

Although the predicted moderation effect was supported, a limitation of the present study is that participants’ sociosexual orientation and their strength of mating motivation were not directly measured, due to considerations of demand characteristics. We were concerned that the filling out these measures may enable the participants to guess the real purpose of the experiment despite the cover story. Therefore, it remains unanswered whether changes in mating motivation strength were responsible for the moderation effect of relationship experience. It is also unclear whether findings from the study are only limited to men who are less open to unrestricted sex. These questions are important in order to reveal the underlying mechanism of the moderation effect of relationship experience in the effect of mating induction on risk-taking and need to be examined by further examination. Also, the current study only used a financial decision task to evaluate individuals’ risk-taking, and replication using different risk-taking tasks in the future studies is encouraged. The current study used a Chinese sample, considering the social learning has been tied to the mechanism of women’s preference of mated over unmated men (Mesoudi et al., 2015) and has a cultural difference as Chinese are found to display stronger social learning than Westerners did in past study (Mesoudi et al., 2015), it is possible that the moderating role of romantic relationship experience on the effectiveness of mating induction may be stronger among people living in East Asian than those in Western societies. This potential cultural difference warrants further examination.

The current study demonstrated that having romantic relationship experience will decrease the effect of mating motivation on man’s financial risk-taking. The current study indicates important real-world implications. Advertising industry and practitioners of persuasion should take into consideration of their targets’ relationship experience to enhance the effectiveness of their persuasive messages, especially in the context of mating and financial decisions. For example, for financial companies and gambling industries, they may consider work with dating service business to better target appropriate consumers (young men without relationship experience) of their high-risk high-return products. The current research also reveals the importance of including men’s past romantic relationship experience in future mating-related research.

## Data Availability Statement

The raw data supporting the conclusions of this article will be made available by the authors, without undue reservation.

## Ethics Statement

The studies involving human participants were reviewed and approved by Institutional Review Board, Department of Psychology, Renmin University of China. The patients/participants provided their written informed consent to participate in this study.

## Author Contributions

TL contributed to the design of study 1, wrote the first draft of the manuscript and contributed to manuscript revision. ZW contributed to the design of study 2, collected data for study 2, conducted statistical analysis and revised the manuscript. AZ contributed to statistical analysis, results visualization, manuscript reviewing and editing. XZ contributed to conception and design of study 1, collected data for study 1. CX is the corresponding author, responsible for designing and correcting the research protocol including conceptualization, methodology, also in charge of manuscript reviewing, editing, supervision, and the whole project administration.

## Funding

This research was supported by the Fundamental Research Funds for the Central Universities, and the Research Funds of Renmin University of China [20XNQ036].

## Conflict of Interest

The authors declare that the research was conducted in the absence of any commercial or financial relationships that could be construed as a potential conflict of interest.

## Publisher’s Note

All claims expressed in this article are solely those of the authors and do not necessarily represent those of their affiliated organizations, or those of the publisher, the editors and the reviewers. Any product that may be evaluated in this article, or claim that may be made by its manufacturer, is not guaranteed or endorsed by the publisher.

## Supplementary Material

The Supplementary Material for this article can be found online at: https://www.frontiersin.org/articles/10.3389/fpsyg.2021.787686/full#supplementary-material
