# Supplementary material for: The Effectiveness of Mating Induction on Men’s Financial Risk-Taking: Relationship Experience Matters
Source: Front Psychol. 2022 Jan 11;12:787686. doi: 10.3389/fpsyg.2021.787686 (PMC8787341; doi:10.3389/fpsyg.2021.787686)
Supplement: Supplementary file 1 [file Table_1.docx]

Supplementary Material

# Supplementary Material for Financial Risk-Taking

There are 10 investment questions below, and each question has two investment schemes. The results of the two investment schemes are as follows. Please mark your preference for the two investment schemes on the 5-point scale. (1 indicates that you like A very much, 2 indicates that you prefer A more, 3 indicates that you have the same preference for the two schemes, 4 indicates that you prefer B more, and 5 indicates that you like B very much).

**Project 1**

Scheme A: Get 45 yuan with 100% probability; Scheme B: Get 50 yuan with 90% probability

Scheme A 1 2 3 4 5 Scheme B

**Project 2**

Scheme A: Get 80 yuan with 100% probability; Scheme B: Get 100 yuan with 80% probability

Scheme A 1 2 3 4 5 Scheme B

**Project 3**

Scheme A: Get 350 yuan with 100% probability; Scheme B: Get 500 yuan with 70% probability

Scheme A 1 2 3 4 5 Scheme B

**Project 4**

Scheme A: Get 1200 yuan with 100% probability; Scheme B: Get 2000 yuan with 60% probability

Scheme A 1 2 3 4 5 Scheme B

**Project 5**

Scheme A: Get 2500 yuan with 100% probability; Scheme B: Get 5000 yuan with 50% probability

Scheme A 1 2 3 4 5 Scheme B

**Project 6**

Scheme A: Get 3200 yuan with 100% probability; Scheme B: Get 8000 yuan with 40% probability

Scheme A 1 2 3 4 5 Scheme B

**Project 7**

Scheme A: Get 3000 yuan with 100% probability; Scheme B: Get 10000 yuan with 30% probability

Scheme A 1 2 3 4 5 Scheme B

**Project 8**

Scheme A: Get 2400 yuan with 100% probability; Scheme B: Get 12000 yuan with 20% probability

Scheme A 1 2 3 4 5 Scheme B

**Project 9**

Scheme A: Get 1500 yuan with 100% probability; Scheme B: Get 15000 yuan with 10% probability

Scheme A 1 2 3 4 5 Scheme B

**Project 10**

Scheme A: Get 1000 yuan with 100% probability; Scheme B: Get 20000 yuan with 5% probability

Scheme A 1 2 3 4 5 Scheme B
